# Pattern of choroidal thickness in early-onset high myopia

**DOI:** 10.3389/fmed.2023.1156259

**Published:** 2023-07-18

**Authors:** Zhaoxin Jiang, Aohan Hou, Ting Zhang, Yanting Lai, Li Huang, Xiaoyan Ding

**Affiliations:** State Key Laboratory of Ophthalmology, Guangdong Provincial Key Laboratory of Ophthalmology and Visual Science, Provincial Clinical Research Center for Ocular Diseases, Zhongshan Ophthalmic Center, Sun Yat-sen University, Guangzhou, China

**Keywords:** high myopia, early onset, choroidal thickness, familial exudative vitreoretinopathy, Stickler syndrome

## Abstract

**Purpose:**

To explore the etiology and choroidal thickness (ChT) pattern in children with early-onset high myopia (eoHM).

**Methods:**

Sixty children with eoHM and 20 healthy controls were enrolled in this study between January 2019 and December 2021. All children underwent comprehensive ophthalmologic examinations including swept-source optical coherence tomography. ChT was measured in the subfoveal region and at 1000 μm and 2,500 μm nasal, temporal, superior, and inferior to the fovea.

**Results:**

Overall, 120 eyes of 60 children with eoHM were examined (mean spherical equivalent, −8.88 ± 3.05 D; mean axial length, 26.07 ± 1.59 mm). Simple high myopia (SHM), familial exudative vitreoretinopathy (FEVR), and Stickler syndrome (STL) were the most frequent etiologies of eoHM and were included in further ChT analysis. Adjusted the effect of SE, multivariate regression analysis showed that children with SHM had thinnest ChT at N_2500_ and I_2500_ among the subgroups (*p* = 0.039, *p* = 0.013). FEVR group showed thinner ChT at T_2500_ (*p* = 0.023), while STL patients exhibited thin ChT at all locations.

**Conclusion:**

This study revealed that SHM, STL and FEVR was the most frequent etiology, and showed a distinctive pattern of ChT. Asymmetric nasal ChT thinning is a distinctive biomarker for SHM, asymmetric temporal ChT thinning might serve as a biomarker for FEVR, and symmetric diffuse thinning is more common in STL. These ChT patterns may provide a convenient, fast, and noninvasive strategy to differentiate the potential etiology of eoHM.

## Introduction

Early-onset high myopia (eoHM), usually defined as high myopia that occurs before 7–10 years of age, accounts for 0.2–0.4% in the general population (1–4). eoHM is a concerning health issue for children as those who develop myopia at an early age have a greater risk of developing pathological myopia by adulthood (5, 6). However, the etiology and pathogenesis remains unclear.

In addition, eoHM may be a specific indicator of ocular or systemic abnormality. Marr et al. reported that only 8% of children had simple high myopia (SHM) while 38% of children had high myopia with one or more ocular condition (s) (3). Stickler syndrome (STL) is a systemic disorder of collagen and the leading cause of retinal detachment in childhood. Fincham et al. reported prevalence of high myopia in 76–80% STL patients. Familial exudative vitreoretinopathy (FEVR) is characterized by incomplete vascularization of the peripheral retina that can cause progressive vision loss (7). High myopia in FEVR is easily mistaken diagnosed as simple high myopia with non-specific vitreoretinopathies (8, 9). Therefore, the diagnosis of SHM could only be made by a process of elimination. As the pathogenesis, treatment and prognosis are distinctive, it is particularly important to distinguish the etiology of eoHM.

The choroid is a vascular structure that supports the metabolic needs of the retinal pigment epithelium and outer retina (10). A marked thinning of the choroid has been demonstrated in studies examining highly myopic adults (11, 12). As myopia begins most commonly in childhood, there have been limited data on the choroidal thickness (ChT) profiles of high myopia in children (13). Thus, this study aimed to investigate the etiology and respective ChT pattern of eoHM, and to develop an OCT based marker for the fast differential diagnose of it.

## Methods

### Study population

Sixty consecutive children with eoHM who visited the Zhongshan Ophthalmic Center between January 2019 and December 2021 were enrolled in the study. Twenty age-matched children with bilateral spherical equivalent (SE) between −0.5 D to +2.5 D and without any ocular diseases, served as the control. This study was performed in accordance with the tenets of the Declaration of Helsinki and approved by the Medical Ethics Committee of Zhongshan Ophthalmic Center, Sun Yat-sen University. Informed consent was obtained from all guardians of the children aged <18 years.

eoHM was defined based on previous studies (2, 14). Briefly, children aged 5–10 years with bilateral myopia of six diopters SE or more and those aged <5 years with bilateral myopia of four diopters SE or more were included in this study. Final diagnosis was provided based on the clinical features and genetic results.

Comprehensive ophthalmologic examinations were performed in each child, including best-corrected visual acuity, intraocular pressure, axial length (AL), slit lamp biomicroscopy, ultra-widefield scanning laser ophthalmoscopy (UWF-SLO, Optos California; Optos, PLC, Dunfermline, Scotland), and swept-source OCT (SS-OCT, VG200D, SVision Imaging, Ltd., Henan, China). Refractions were determined 30 min after instillation of three drops of cyclopentolate in children older than 6 years, and 3 days after administration of atropine ointment in children less than 6 years old. Systemic abnormalities, birth history, and family history of ocular abnormalities of each child were recorded.

### Choroidal thickness measurements

**Figure 1:**
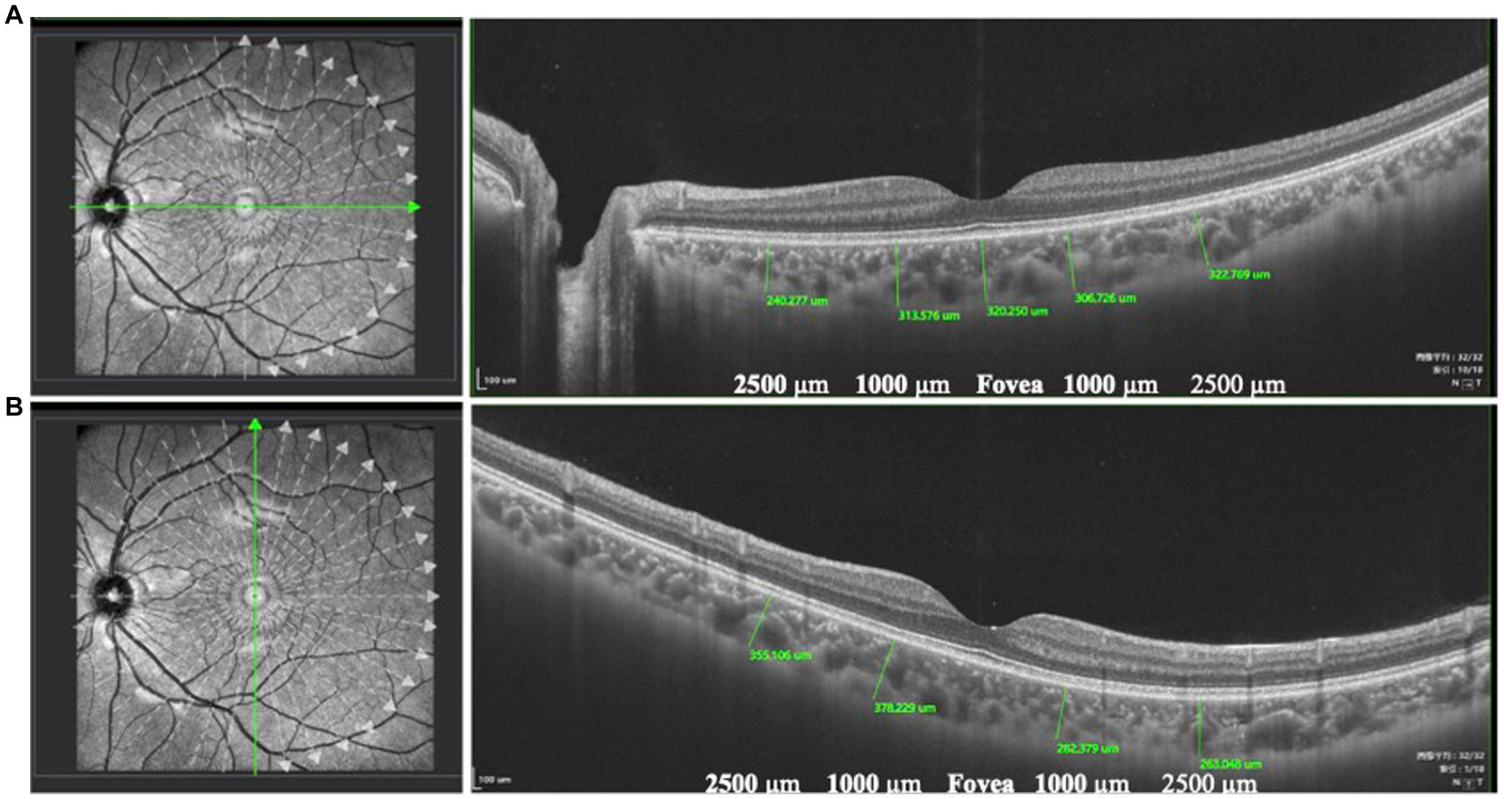
The measurement of ChT. ChT was the distance between the outer portion of the hyperreflective line corresponding to the RPE and the inner surface of the sclera. ChT was measured at the fovea and at 1000 μm and 2,500 μm nasal, temporal **(A)**, superior, and inferior to the fovea **(B)**.

All children with dilated pupils were examined using SS-OCT (VG200D; SVision Imaging, Ltd., Henan, China). The OCT scanning protocol included a length of 12 mm with 18 equal radial meridian scans centered on the fovea. The images were viewed and measured using built-in calipers in the software. ChT is the distance between the outer portion of the hyperreflective line corresponding to the retinal pigment epithelium and the inner surface of the sclera (15, 16). ChT was measured at the fovea, and at 1000 μm and 2,500 μm nasal, temporal, superior, and inferior to the fovea (14) (Figure 1). All measurements were performed by two experienced retinal specialists (H. A. and Z. T.). The intraclass correlation efficient (ICC) values of intra-and interobserver agreement were ranged from 0.92 to 0.96 for all parameters (*p*<0.001). The reported data represents the average of six measurements, with each measurement being taken by two specialists who each took three measurements. A topographic map of ChT was obtained according to the color code provided by the built-in software (warm colors indicate a thick choroidal structure while cold colors indicate a thin choroidal structure) (17).

### Statistical analyses

All data were collected in an electronic database and crosschecked for error. Continuous variables were presented as the mean ± standard deviation. Data distribution was examined using the Kolmogorov–Smirnov test. Intergroup differences were assessed using t-tests or analysis of variance when the data were normally distributed, while the Mann–Whitney *U* test or Kruskal–Wallis test was used for skewed distributions. Post-hoc tests were performed using least significant difference method. Parameters with significance in univariate analysis were retested using multivariate analysis. Multivariate regression models were used for multivariate adjustment of confounders. SPSS (version 16.0; SPSS, Chicago, Illinois, United States) was used to perform statistical analyses. Statistical significance was set at *p* < 0.05.

## Results

### Demography and etiology of eoHM

Sixty children with eoHM were enrolled in this study and a total of 120 eyes were examined. The mean age of the children was 4.66 ± 2.25 years (range: 3 to 10 years) and 39 (64.41%) of them were male. Family history and/or the presence of ocular abnormalities in first-degree relatives was found in 29 (48.33%) children.

Based on the clinical and genetic features, 14 cases (23.3%) were confirmed as familial exudative vitreoretinopathy (FEVR, indicated by the presence of abnormal retinal features including peripheral avascular area, vascular straightening, and increased branching (18, 19)) and 10 cases (16.7%) were diagnosed as STL (indicated by the presence of vitreous membrane (20, 21)) (Table 1). SHM was diagnosed in 20 (33.3%) children. The other 16 cases were genetically identified as retinitis pigmentosa (*n* = 4), congenital stationary night blindness (*n* = 4), Leber congenital amaurosis (*n* = 1), Cooffin Siris syndrome (*n* = 1), Knobloch syndrome (*n* = 1), Bohring-Opitz syndrome (*n* = 1), Donnai Barrow syndrome (*n* = 1), and retinopathy of prematurity (*n* = 3).

**Table 1:** Etiology of eoHM.

Abnormality detected	Number of patients (%)
Simple high myopia with no associated conditions	20 (33.33)
*Ocular conditions*	
Familial exudative vitreoretinopathy	14 (23.33)
Congenital stationary night blindness	4 (6.67)
Retinitis pigmentosa	4 (6.67)
Leber congenital amaurosis	1 (1.67)
Retinopathy of prematurity	3 (0.05)
*Systemic conditions*	
Stickler’s syndrome	10 (16.67)
Bohring-Opitz syndrome	1 (1.67)
Cooffin siris syndrome	1 (1.67)
Donnai Barrow syndrome	1 (1.67)
Knobloch syndrome	1 (1.67)
Total	60 (100)

### Clinical characteristics of eoHM

The mean SE was −8.88 ± 3.05 D (range: −18.75 to −4.0 D) and the mean AL was 26.07 ± 1.59 mm (range: 21.81–33.05 mm). There were no obvious abnormalities in anterior segment evaluations in all children. In SS-OCT examination, ten eyes with eoHM were excluded because of poor image quality (*n* = 6) and retinal detachment (*n* = 4). ChT of the remaining 110 eyes was analyzed (Table 2). Compared to the normal controls, eyes with eoHM showed a significantly thinner choroid at all locations (*p* < 0.0001). Among the locations, ChT was the thinnest at N_2500_ in both control (188.82 ± 64.00 μm) and highly myopic eyes (88.97 ± 55.71 μm).

**Table 2:** Clinical Characteristics of controls and eoHM.

	Control (*n* = 20)	eoHM (*n* = 60)	*p* value^*^	Subgroups in eoHM			*p* value^#^
				SHM (*n* = 20)	STL (*n* = 10)	FEVR (*n* = 14)	
Age, y	5.47 (1.81)	4.66 (2.25)	0.0709	4.35 (1.44)	5.40 (2.56)	5.93 (2.81)	0.1541
AL, mm	23.00 (1.43)	26.07 (1.59)	<0.0001	26.11 (1.03)	27.12 (1.51)	25.84 (1.75)	0.0401
SE, D	1.52 (1.06)	−8.88 (3.05)	<0.0001	−8.36 (1.81)	−12.22 (3.83)	−7.99 (2.44)	0.0002
BCVA, logMAR	0.15 (0.20)	0.55 (0.58)	0.0002	0.47 (0.55)	0.75 (0.90)	0.54 (0.42)	0.3320
ChT, um							
Sub-foveal	331.73 (75.23)	183.96 (91.10)	<0.0001	189.75 (79.85)	147.69 (54.88)	189.62 (71.80)	0.2753
N_2500_	188.82 (64.00)	88.97 (55.71)	<0.0001	62.67 (35.19)	89.31 (45.60)	133.81 (62.37)	0.0001
N_1000_	286.09 (69.93)	147.60 (76.20)	<0.0001	141.78 (57.64)	126.44 (61.25)	165.57 (68.30)	0.2754
T_1000_	360.23 (64.11)	198.36 (91.38)	<0.0001	213.22 (213.22)	175.50 (58.79)	182.75 (59.82)	0.3147
T_2500_	354.91 (63.11)	226.38 (98.06)	<0.0001	252.56 (101.12)	195.81 (83.94)	186.42 (62.18)	0.0146
S_2500_	321.06 (60.61)	194.29 (82.91)	<0.0001	186.25 (89.07)	109.00 (48.94)	236.00 (62.07)	0.0002
S_1000_	325.29 (80.04)	184.52 (86.32)	<0.0001	182.55 (64.78)	131.00 (61.13)	199.37 (52.56)	0.0104
I_1000_	333.05 (70.96)	165.19 (73.81)	<0.0001	159.00 (70.83)	144.40 (54.88)	178.17 (50.91)	0.0759
I_2500_	324.38 (76.05)	165.29 (62.42)	<0.0001	133.57 (29.48)	140.25 (73.13)	191.88 (57.86)	0.1238
Features							
Symmetry				Asymmetric	Symmetric	Asymmetric	
Location				Nasal-predominant thinning	Global thinning	Temporal-predominant thinning	

The data shown as Mean (SD). ^*^ Compared between eoHM and control. ^#^ Compared among 3 subgroups. eoHM, early onset high myopia; SHM, simple high myopia; STL, Stickler syndrome; FEVR, familial exudative vitreoretinopathy.

### Choroidal thickness pattern in subgroups of eoHM

Pattern of ChT in patients with SHM, STL, and FEVR were further analyzed (Table 2). Other subgroups with fewer than five cases were not included, due to the small sample size. There were no statistical differences in the mean age (*p* = 0.1541) of these subgroups. The mean SE was −12.22 ± 3.83 D in STL, which was significantly lower than that of SHM (−8.36 ± 1.81 D, *p* < 0.0001) and FEVR subgroup (−7.99 ± 2.44 D, *p* = 0.0003). Consistently, the STL also showed significantly longer AL than the SHM and FEVR subgroups (*p* = 0.0178 and *p* = 0.0013, respectively).

**Figure 2:**
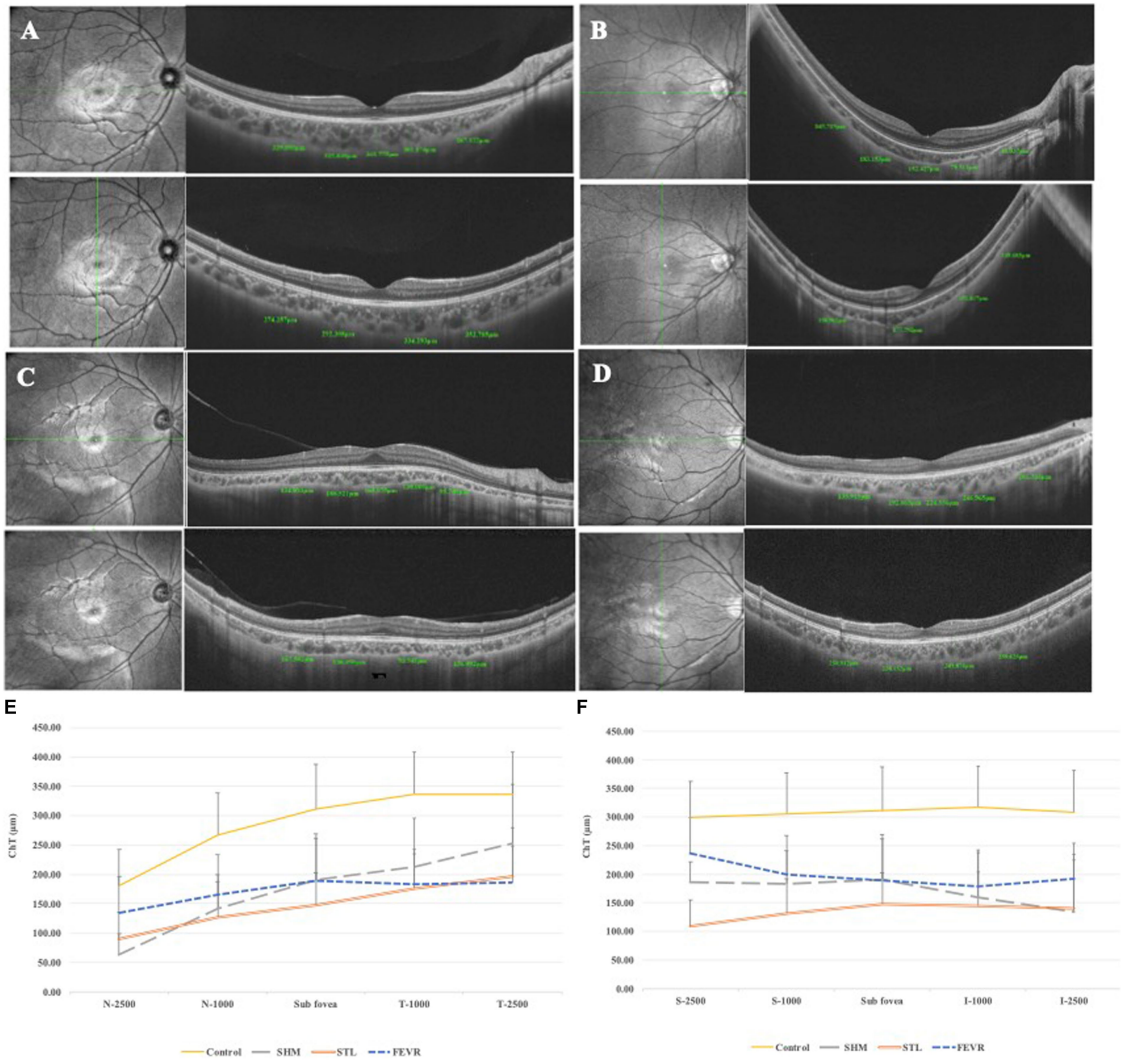
ChT of eoHM and controls. **(A)** Control, **(B)** A patient with SHM showed obvious thin ChT as 41.037 μm at N_2500_, **(C)** A patient with STL showed thin ChT both in the horizontal and vertical sections, **(D)** A patient with FEVR showed obvious thin ChT as 135.92 μm at T_2500_. **(E)** Horizontal section through the fovea, and **(F)** Vertical section through the fovea. SHM showed the thinnest ChT at N_2500_, FEVR showed the thinnest ChT at T_2500_, while STL showed thin ChT at all locations.

**Figure 3:**
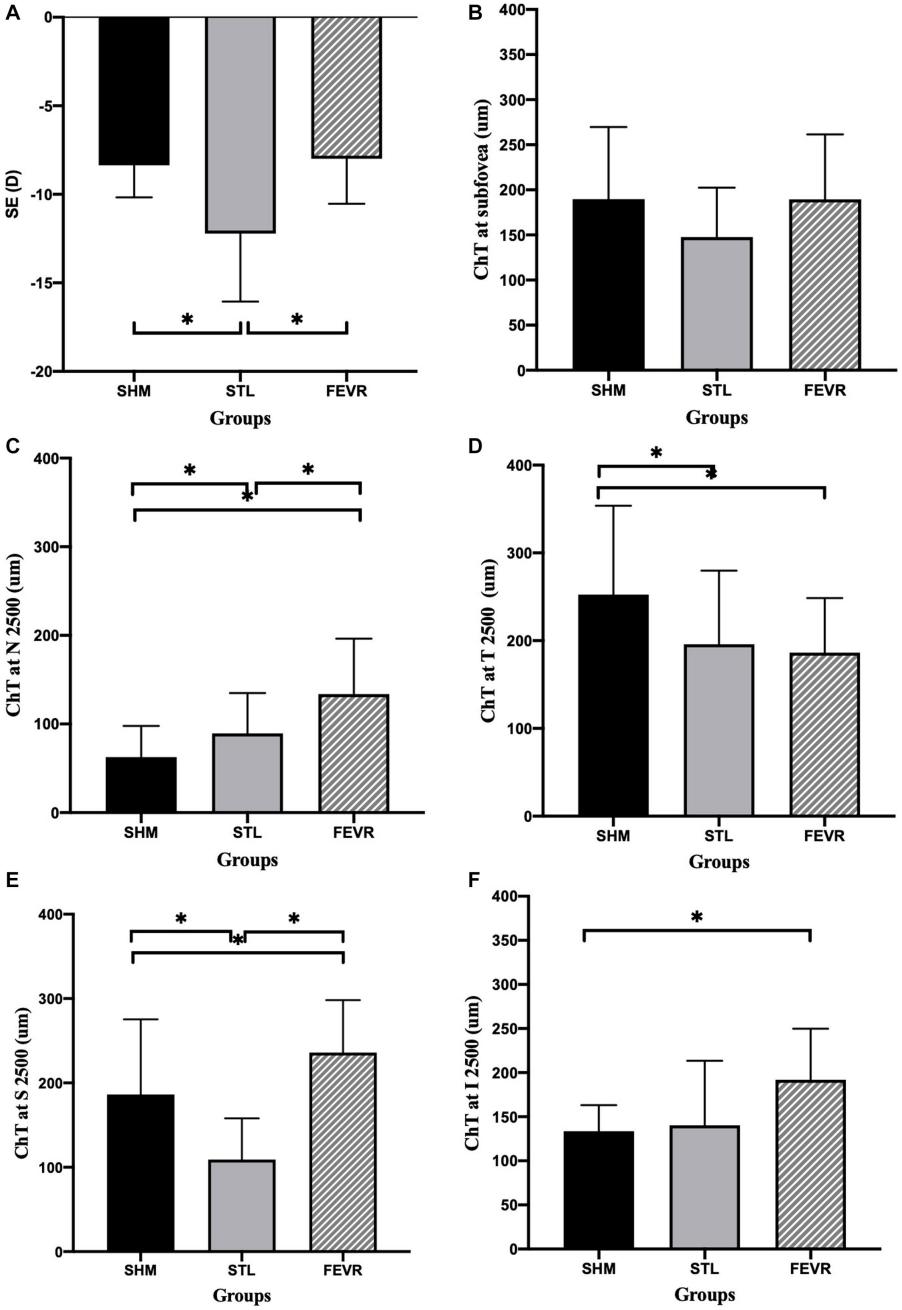
ChT at different locations among subgroups of eoHM. **(A)** STL showed significantly higher SE than that of in SHM and FEVR groups, **(B)** ChT at the subfoveal region was similar among subgroups, **(C–F)** At N_2500_, SHM showed the thinnest ChT among subgroups. At T_2500_, FEVR showed the thinnest ChT. At S_2500_, STL showed the thinnest ChT. At I_2500_, both SHM and STL showed obvious thinning of ChT.

Interestingly, the ChT varied significantly across subgroups (Figure 2). The parameters for 2,500 μm seemed more meaningful than that for 1,000 μm, as ChT at N_2500_ and T_2500_ showed significant difference among the subgroups of eoHM (*p* < 0.05), while ChT at N_1000_ and T_1000_ were similar among them (*p* > 0.05). At N_2500_, SHM showed the thinnest ChT among the subgroups, whereas FEVR showed the thickest (*p* = 0.0001). At T_2500_, by contrast, SHM showed the thickest ChT (*p* = 0.0146), whereas FEVR showed the thinnest ChT. At S_2500_, STL showed the thinnest ChT among the subgroups (*p* = 0.0002). At I_2500_, both SHM and STL showed obvious thinning of the ChT (*p* = 0.1238). The mean subfoveal choroid thickness (SFCT) was similar among the subgroups (*p* = 0.2753; Figure 3).

Univariate analysis revealed that SE and AL were significantly associated with ChT at all locations (*p* < 0.05 and *p* < 0.05, Table 3). To adjust for the effect of SE, multivariate regression analysis was performed with ChT as a dependent variable and SE as an independent variable. After adjusting for SE, the SHM subgroup showed significantly thinner ChT than the other subgroups at N_2500_ and I_2500_ (*p* = 0.039 and *p* = 0.013). FEVR showed a significantly thinner ChT than the other subgroups at T_2500_ (*p* = 0.023). However, there was no significant difference in ChT at S_2500_ among the subgroups (Table 4).

**Table 3:** Univariate regression analysis of associations between AL, SE, and ChT in eoHM.

Location	Ocular factors	Univariate analysis		
		Unstandardized beta coefficient	95% confidence interval	*p* value
Subfoveal	AL, mm	−32.65	(−40.38, -24.91)	<0.001
	SE, D	11.69	(8.93,14.45)	<0.001
N_2500_	AL, mm	−22.38	(−28.01, −16.75)	0.001
	SE, D	7.69	(5.56,9.81)	0.016
N_1000_	AL, mm	−29.64	(−36.35, −22.92)	<0.001
	SE, D	10.66	(8.19,13.13)	<0.001
T_1000_	AL, mm	−22.15	(−40.86, −23.44)	0.003
	SE, D	11.99	(8.86,14.91)	0.012
T_2500_	AL, mm	−22.11	(−32.16, −12.07)	<0.001
	SE, D	9.7	(6.26,13.14)	<0.001
S_2500_	AL, mm	−22.61	(−31.87, −13.35)	<0.001
	SE, D	10.82	(7.83,13.80)	<0.001
S_1000_	AL, mm	−32.32	(−40.30,24.34)	<0.001
	SE, D	12.39	(9.62,15.16)	<0.001
I_1000_	AL, mm	−35.82	(−42.74,-28.90)	<0.001
	SE, D	13.77	(11.39,16.15)	<0.001
I_2500_	AL, mm	−32.64	(−40.79,24.49)	<0.001
	SE, D	12.90	(10.05,15.75)	<0.001

**Table 4:** Multivariate analysis of associations between SE and ChT in eoHM.

Location	Ocular factors	Multivariate analysis		
		Unstandardized beta coefficient	95% confidence interval	*p* value
N_2500_	SE, D	6.75	(2.64, 10.86)	0.016
	SHM vs. others	−48.53	(−94.65, −2.61)	0.039
	FEVR vs. others	20.29	(−26.62, 67.21)	0.393
	Stickler vs. others	2.12	(−59.65, 63.90)	0.946
T_2500_	SE, D	5.76	(−1.50, 13.01)	0.001
	SHM vs. others	−25.26	(−106.11, 55.59)	0.536
	FEVR vs. others	−97.31	(−180.60, −14.02)	0.023
	Stickler vs. others	−64.62	(−173.84, 44.61)	0.243
S_2500_	SE, D	8.72	(2.75, 14.68)	0.005
	SHM vs. others	−30.17	(−101.90, 41.55)	0.402
	FEVR vs. others	20.53	(−45.26, 86.31)	0.534
	Stickler vs. others	−73.37	(−163.68, 16.94)	0.109
I_2500_	SE, D	9.51	(3.64, 15.38)	0.002
	SHM vs. others	−91.17	(−161.95, −20.38)	0.013
	FEVR vs. others	−28.94	(−93.51, 5.64)	0.373
	Stickler vs. others	−44.46	(−133.14, 44.23)	0.319

**Figure 4:**
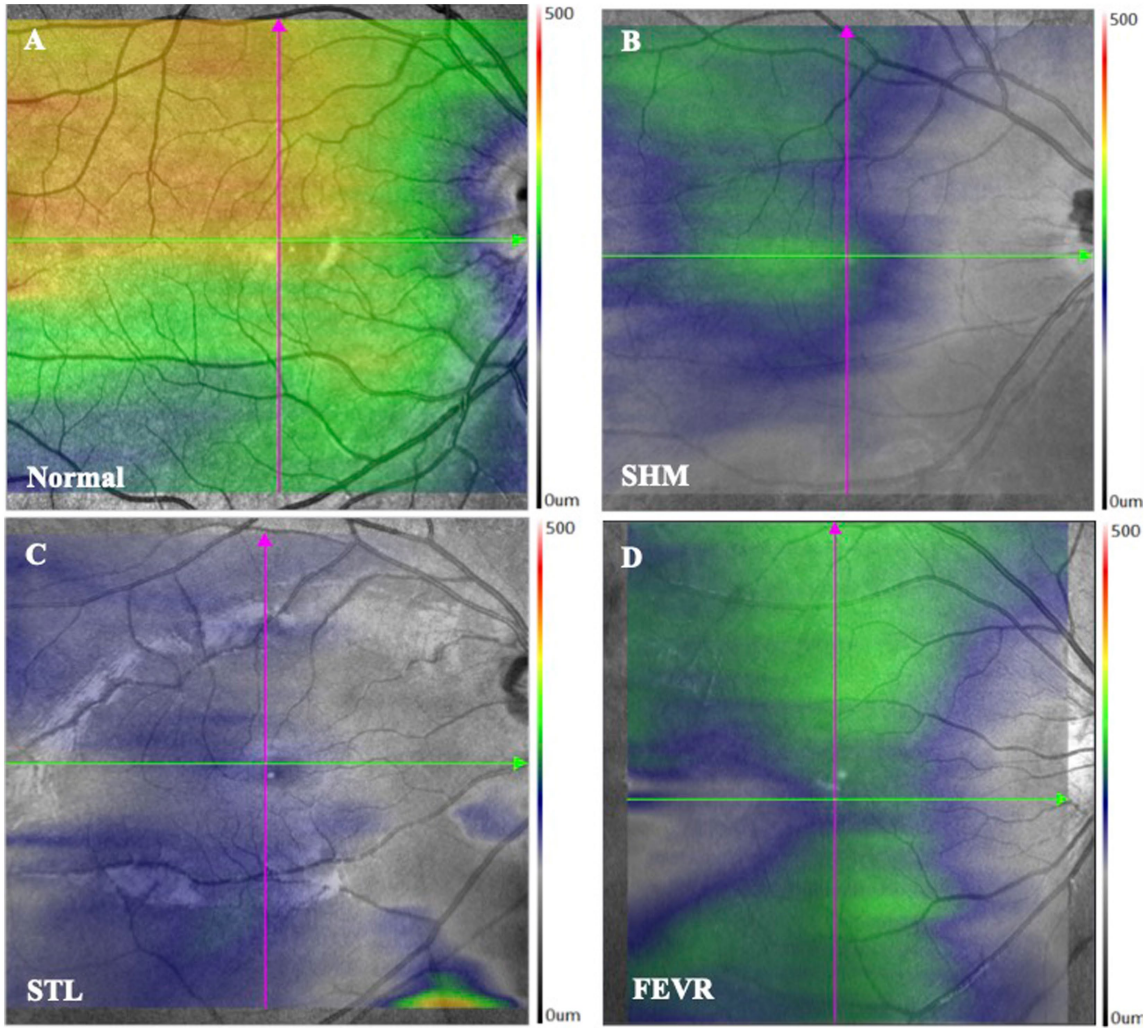
ChT topography of controls and eoHM. **(A)** Control, **(B)** A patient with SHM showed asymmetrical and nasal-predominant thinning pattern, **(C)** A patient with STL showed symmetrical global thinning pattern, and **(D)** A patient with FEVR showed asymmetrical temporal-predominant thinning pattern of ChT.

Taken together, the symmetry and pattern of ChT were significantly distinctive in SHM, STL, and FEVR. The pattern of ChT in SHM is asymmetrical with nasal-predominant thinning. By contrast, eyes with FEVR harbored the thinnest ChT on the temporal side and the thickest ChT on the nasal side, which could be referred to as asymmetrical temporal-predominant thinning pattern. In STL, the ChT pattern showed symmetrical global thinning (Figure 4).

## Discussion

The diagnosis of the etiology of eoHM is still challenging. eoHM may be the first clue to conditions such as STL, FEVR or Marfan syndrome. Early diagnosis and treatment of certain conditions linked with myopia at an otherwise asymptomatic stage may prolong vision and/or life. In the present study, the ChT of eoHM was analyzed and revealed distinctive patterns with different etiologies. SHM showed the thinnest ChT at the nasal and inferior regions to the fovea. FEVR displayed an asymmetrical temporal-predominant thinning pattern of ChT, whereas STL showed thin ChT at all measured locations. This distinctive distribution pattern of ChT may provide a convenient, fast, and noninvasive strategy to differentiate the etiology of eoHM.

### ChT pattern of SHM was consistent with previous studies

In normal eyes, a pattern of thinner choroid nasally compared to temporally, and inferiorly compared to superiorly, has been a relatively consistent finding in studies (22, 23). Studies showed that the thinning of the ChT in myopia was more pronounced inferiorly and nasally than superiorly and temporally (24). Read et al. suggested the potential presence of asymmetries in ocular growth patterns, and Moderiano et al. showed that there are regional differences in the sensitivity of the human choroid to defocus, and these differences may be due to regional variations in choroidal blood flow distribution (25, 26). In highly myopic eyes, Yokio et al. showed that the ChT was thinnest at the inferior and nasal locations, followed by the superior and temporal locations. In addition, ChT at N_2500_ was less than 60 μm in 31 (76%) of 41 highly myopic eyes, while in none (0/1463) of the control group, and suggested that the <60 μm ChT at N_2500_ as a potentially useful cutoff value for high myopia diagnosis (14). In the current study, the ChT in eoHM was 62.67 μm at N_2500_, which is consistent with previous reports. Thus, asymmetric nasal-predominant thinning of ChT could serve as a distinctive biomarker for SHM in children.

### ChT pattern of FEVR was reported for the first time

FEVR is characterized by incomplete or vascularization of the peripheral retina, leading to including retinal ischemia and tractional retinal detachments. Our previous work revealed peripherally retinal thinning in all FEVR eyes (19). However, reports on ChT pattern in FEVR are still lacking. Yonekawa et al. showed that SFCT was as thin as 216 ± 64 μm in 39 FEVR cases but no details were provided for other regions (27). Similarly, the mean SFCT as 189 ± 71.80 μm was reported in the FEVR group in the current study. To the best of our knowledge, ChT pattern of FEVR was reported for the first time and revealed the thinnest ChT among subgroups at T_2500_. Studies showed that peripheral retina plays an important role in guiding the growth of ocular components during emmetropization, and the extensive peripheral atrophy of retina and choroid alter the response of the sclera to growth signals (28, 29). Thus, the asymmetric temporal-predominant thinning of ChT may be served as an indicator for FEVR.

### STL exhibited global thinning of ChT

In current study, children with STL showed highest mean SE (12.22 ± 3.83 D) and longest axial length (27.12 ± 1.51 mm). This global thinning ChT pattern seems to be in part related to the scleral remodeling and axial elongation (30). Stickler syndrome is a hereditary connective tissue disorder of fibrillar collagen. Studies of myopia have showed the association between scleral thinning and axial elongation (31, 32). On the other hand, Xerri et al. showed a significantly thicker ChT in STL adults compared with controls matched for axial length (33). By contrast, current study showed that ChT in STL children was thinner than in SHM group in almost all areas. Popkin et al. reported that high myopia associated with STL is typically present at birth and has a non-progressive course, while SHM begins in childhood and progresses commonly with age (34). Thus, it will be interesting to assess the changes of refractive errors and ChT base on a long-term follow-up.

Our study has some limitations. First, as the study was hospital-based, no conclusion could be drawn concerning the prevalence and association of high myopia in the wider pediatric population. Second, the sample size in the current study was limited owing to the rarity of eoHM, which made it difficult to recruit a larger cohort. Third, there was no long-term follow-up assessing the changes in ChT of the patients. Nonetheless, with a moderately large population, the current study provides a new and convenient biomarker for fast screening that is worth exploring in future case–control studies.

## Conclusion

Current study showed that SHM, STL, and FEVR were the most frequent diseases associated with eoHM. These eoHM subtypes show a distinctive pattern of ChT. SHM harbored the thinnest ChT at the nasal and inferior regions, while FEVR showed the thinnest ChT at the temporal region of the fovea, and the STL group showed thinned ChT in all areas. The ChT pattern may provide a new biomarker for the fast screening of eoHM and facilitate diagnostic genetic testing. Further investigations with larger sample sizes are required to confirm our observations.

## Data availability statement

The data analyzed in this study is subject to the following licenses/restrictions: all the data used to support the findings of this study are included within the article and are available from corresponding author by a reasonable request. Requests to access these datasets should be directed to XD, dingxiaoyan@gzzoc.com.

## Ethics statement

This study was approved by the Medical Ethics Committee of Zhongshan Ophthalmic Center, Sun Yat-sen University. Written informed consent to participate in this study was provided by the participants’ legal guardian/next of kin.

## Author contributions

XD, ZJ, and AH contributed to conception and design of the study. ZJ, AH, TZ, YL, and LH organized the database. AH performed the statistical analysis. XD and ZJ wrote the first draft of the manuscript. All authors contributed to the article and approved the submitted version.

## Funding

This study was supported by Science and Technology Program Guangzhou, China (201803010031; Guangzhou, Guangdong, China), the National Natural Science Foundation of China (nos. 81900896 and 82000910). The sponsors and funding organizations had no role in the design or conduct of this research.

## Conflict of interest

The authors declare that the research was conducted in the absence of any commercial or financial relationships that could be construed as a potential conflict of interest.

## Publisher’s note

All claims expressed in this article are solely those of the authors and do not necessarily represent those of their affiliated organizations, or those of the publisher, the editors and the reviewers. Any product that may be evaluated in this article, or claim that may be made by its manufacturer, is not guaranteed or endorsed by the publisher.
